# Physiological and Biochemical Characterization of a Novel Nicotine-Degrading Bacterium *Pseudomonas geniculata* N1

**DOI:** 10.1371/journal.pone.0084399

**Published:** 2014-01-08

**Authors:** Yanghui Liu, Lijuan Wang, Kaiming Huang, Weiwei Wang, Xueling Nie, Yi Jiang, Pengpeng Li, Shanshan Liu, Ping Xu, Hongzhi Tang

**Affiliations:** 1 State Key Laboratory of Microbial Metabolism, and School of Life Sciences & Biotechnology, Shanghai Jiao Tong University, Shanghai, People's Republic of China; 2 School of Environmental Science and Engineering, Shanghai Jiao Tong University Shanghai, People's Republic of China; 3 Shanghai Nuclear Engineering Research & Design Institute, Shanghai, People's Republic of China; Massachusetts Institute of Technology, United States of America

## Abstract

Management of solid wastes with high nicotine content, such as those accumulated during tobacco manufacturing, poses a major challenge, which can be addressed by using bacteria such as *Pseudomonas* and *Arthrobacter*. In this study, a new species of *Pseudomonas geniculata*, namely strain N1, which is capable of efficiently degrading nicotine, was isolated and identified. The optimal growth conditions for strain N1 are a temperature of 30°C, and a pH 6.5, at a rotation rate of 120 rpm min^−1^ with 1 g l^−1^ nicotine as the sole source of carbon and nitrogen. Myosmine, cotinine, 6-hydroxynicotine, 6-hydroxy-N-methylmyosmine, and 6-hydroxy-pseudooxynicotine were detected as the five intermediates through gas chromatography-mass and liquid chromatography-mass analyses. The identified metabolites were different from those generated by *Pseudomonas putida* strains. The analysis also highlighted the bacterial metabolic diversity in relation to nicotine degradation by different *Pseudomonas* strains.

## Introduction

Nicotine, a principal pyridine alkaline in tobacco plants, is notorious for its significant contribution to tobacco addiction. However, nicotine is very toxic to humans because it is easily absorbed in the body; its hydrophilic nature contributes to the environmental contamination [1]. Moreover, large quantities of tobacco wastes containing high concentration of nicotine are produced during tobacco manufacturing process. These wastes have been classified as “toxic and hazardous wastes” by European Union Regulations [2]. In addition, the American Medical Association has issued a public strategy strengthening the forcible reduction of nicotine level in tobacco [3]. As an environment-friendly treatment, microbial degradation of nicotine has been considered as a promising method due to its low cost and high efficiency.

In recent years, studies elucidating the mechanisms underlying nicotine degradation by microorganisms have drawn considerable attention. Previously, several bacteria including *Arthrobacter* species [4], *Pseudomonas* species [5]–[8], *Ochrobactrum intermedium* [9], *Rhodococcus* species [10], *Ensifer* species [11], and *Agrobacterium* species [12] have been reported to degrade nicotine. There are three pathways for nicotine metabolism: (1) the methyl pathway, used by some fungi such as *Pellicularia filamentosa*, in which organisms degrade nicotine by demethylation to nornnicotine [13]; (2) the pyridine pathway, in which nicotine degradation begins with the hydroxylation of pyridine ring to generate 6-hydroxynicotine, is commonly used by *Arthrobacter* [4], *Nocardioides* [14] and *Rhodococcus* [10] species; (3) pyrrolidine pathway through which nicotine is initially dehydrogenated at the pyrrolidine ring to form *N*-methylmyosimine, is commonly used by some *Pseudomonas* species [15]–[18].

In this study, a novel strain, *Pseudomonas geniculata* N1, capable of degrading nicotine was isolated. Along with the identification and characterization of this new nicotine-degrading strain, we also determined the optimal conditions for cell growth and nicotine degradation. Compared with other *Pseudomonas* and *Arthrobacter* species, strain N1 exhibited a distinct color change, during its growth with nicotine as the sole source of carbon and nitrogen. The intermediates of strain N1-mediated nicotine degradation were identified by high-performance liquid chromotography (HPLC), ultraviolet (UV) absorption, gas chromatography mass (GC-MS), and liquid chromatography mass (LC-MS) analysis. The data showed that strain N1 decomposes nicotine via a unique pathway, which is different from those reported by *Pseudomonas* strains. This study suggests that the nicotine-degrading bacterium has future potential application on the treatment of the waste generated during tobacco manufacturing. The findings might help further the research for characterizing the molecular mechanisms underlying nicotine degradation by strain N1.

## Materials and Methods

### Chemicals and media

L-(-)-Nicotine (≥99% purity) was purchased from Fluka Chemie GmbH (Buchs, Switzerland). All other chemicals were of analytical grade. The “*nic* medium” was a minimal medium containing 13.3 g K_2_HPO_4_·3H_2_O, 4 g KH_2_PO_4_, 0.2 g MgSO_4_·7H_2_O and 0.5 ml of trace elements solution. L-(-)-Nicotine was added to this minimal medium after filtration sterilization to a final concentration of 1 g l^−1^. The trace elements solution contained: 0.05 g CaCl_2_·2H_2_O, 0.05 g CuCl_2_·2H_2_O, 0.004 g FeSO_4_·7H_2_O, 0.008 g MnSO_4_·H_2_O, 0.1 g Na_2_MoO_4_·2H_2_O, 0.05 g Na_2_WO_4_·2H_2_O, and 0.1 g ZnSO_4_ (per liter of 0.1 mM HCl).

### Strain identification and characterization

After the extraction of genomic DNA by the Wizard Genomic DNA purification kit (Promega Corp., Madison, WI, USA), 16S rRNA gene was amplified by PCR with the universal primer pair of 27F (5′-AGAGTTTGATCCTGGCTCA-3′) and 1492R (5′-GGTTACCTTGTTACGACTT-3′). PCR amplification was carried out with pfu polymerase (Tiangen, Beijing, China) by denaturation at 94°C for 5 min, followed by 30 cycles of 94°C for 30 s, 60°C for 30 s, and 72°C for 3 min. The PCR product was purified for sequence analysis and homology alignment analysis using the BLAST search program (http://www.ncbi.nlm.nih.gov/BLAST.html). A phylogenetic tree was constructed with the neighbor-joining (NJ) method using MEGA 4.1 [19].

A series of experiments were conducted for simultaneously identifying the morphological, physiological and biochemical characteristics of the strain. The morphology was studied using a transmission electron microscope. The physiological and biochemical characteristics such as the utilization of different carbon sources and enzymatic properties were determined by China Center for Type Culture Collection (CCTCC).

### Cell growth and nicotine degradation

Culture temperature, pH, nicotine concentration and rotation rates were studied in order to identify the optimal conditions for cell growth and nicotine transformation. To determine the optimal temperature for cell growth, strain N1 was incubated in minimal medium containing 1 g l^−1^ nicotine at 23°C, 26°C, 30°C, 34°C, and 37°C with the initial pH set at 7.0. The optimal pH was determined by culturing N1 at pH values of 4.5, 5.0, 5.5, 6.0, 6.5, 7.0, and 7.5. The pH was adjusted using 100 mmol phosphorous buffer. Once the optimal temperature and pH were determined, the effect of nicotine concentration (0.5, 1.0, 1.5, and 2.0 g l^−1^) was investigated under optimal temperature and pH. To evaluate the influence of the rotation rate of the reciprocal shaker on nicotine degradation, strain N1 was cultivated at different shaking rates of 0, 120, 180, and 220 rpm. The optimized conditions were then used for subsequent work.

During the incubation period, aliquots of the culture medium were sampled at the pre-determined intervals and analyzed at 600 nm by using a 2100 spectrophotometer (Unic Company, Shanghai). The samples were also preserved at −20°C for HPLC and UV absorption analyses.

### Degradation of nicotine by strain N1

*P*. *geniculata* N1 was cultured under optimal conditions in “*nic* medium”, Luria-Bertani (LB) medium and LB medium with 1 g l^−1^ nicotine, harvested during the late-exponential phase by centrifugation at 6,000× g for 8 min at 4°C, and washed twice by sodium phosphate buffer (100 mM, pH 7.0). Then the cells were suspended in deionized water (OD_600 nm_∼5) for reaction (called resting cells). Resting cells were resuspended in prepared PBS buffer (pH 7.0), and adjusted to OD_600 nm_∼15, with the addition of 10% glycerol, 1 mM DTT and 2.5 mM PMSF. After sonification in the condition of 5 s on, 5 s off, 90 cycles, cell lysates were centrifuged at 12,000 rpm for 20 min, and the supernatant liquid were used for reaction (called crude cells). The nicotine degradation assay was performed at 30°C on a shaker rotating at 180 rpm.

### Identification of metabolites in nicotine degradation

After the “resting cell reaction”, the reaction mixture (1 ml) was evaporated until its dry at 50°C under the reduced pressure, and then dissolved in 200 µl of acetonitrile. The resulting solution was transferred to a vial and dried under nitrogen stream. Samples were analyzed using a GC-MS system (GCD 1800C, Hewlett-Packard) equipped with a flame ionization detector and a 50-m-long J&W DB-5MS column (Folsom, CA, USA) at 140°C. The injection port and detector were set at 260°C and 280°C, respectively. LC-MS analysis was performed by Agilent 1290 (ultra-performance liquid chromatography, UPLC) coupled with an Agilent 6230 electrospray ionization-time-of-flight-mass spectrometry (ESI-TOF-MS) with methanol-0.1% formic acid mixture and H_2_O (95∶5, v v^−1^, 0.5 ml min^−1^). The system was equipped with C18 column (1.8-um thick, 2.1×50 mm; Agilent).

### General analytical techniques

The nicotine present in the culture medium was quantified by HPLC (Agilent 1200 series) equipped with an Eclipse XDB-C18 column (column size, 250×4.6 mm; particle size, 5 µm; Agilent). A mixture of methanol-1 mmol H_2_SO_4_ (5∶95 v v^−1^) was used as the mobile phase, at a flow rate of 0.5 ml min^−1^. Qualitative analysis of nicotine and metabolites was carried out by UV-2500 spectrophotometer (Shimadzu).

### Nucleotide sequence accession number

The nucleotide sequence reported in the present study has been deposited in the GenBank under the accession number JN607239.

## Results

### Isolation and identification of strain N1

The isolated strain N1, which can utilize nicotine as the sole source of carbon and nitrogen, has been deposited at CCTCC under the accession number M2011183. Strain N1 forms small, circular, and convex colonies with neat edges on nicotine agar (Figure S1A). Notably, the colonies were yellow, which is rarely observed in case of nicotine-degrading strains. The strain was identified as a non-spore-forming, gram-negative rod (0.5×1.5 µm) with 2 or 3 flagella at one pole. The image of strain N1 is presented in Figure S1B. Strain N1 could utilize a narrow range of carbon sources such as polychrom, and it grew weakly in glucose, amygdalin, arbutin, and saligenin (Table 1). The physiological and biochemical characteristics, performed at CCTCC, are shown in Tables 1 and 2. Strain N1 incubated at 30°C showed the following characteristics: growth at 5% sodium chloride; positive for catalase and oxidase; positive for arginine dihydrogenase and lysine decarboxylase; negative for ornithine decarboxylase; utilization of citric acid or polychrome as the sole source of carbon for growth; positive for lipase (C14); negative for H_2_S and indole production; and negative for Voges-Proskauer test (Table 2). The characteristics of strain N1 were strikingly similar to those of previously reported *Pseudomonas geniculata* strains [20].

**Table 1:** Utilizations of carbon sources by strain N1.

Tests	Result	Tests	Result
0 Control	−	25 Polychrom	+
1 Glycerol	−	26 Saligenin	w
2 Erythritol	−	27 Cellobiose	−
3 D- arabinose	−	28 Maltose	−
4 L- arabinose	−	29 Lactose	−
5 Ribose	−	30 Melibiose	−
6 D- xylose	−	31 Sucrose	−
7 L- xylose	−	32 Trehalose	−
8 Adonitol	−	33 Synanthrin	−
9 β- methyl -D- xyloside	−	34 Melezitose	−
10 Galactose	−	35 Raffinose	−
11 Glucose	W	36 Starch	−
12 Fructose	−	37 Glycogen	−
13 Seminose	−	38 Xylitol	−
14 Sorbose	−	39 Gentiobiose	−
15 Rhamnose	−	40 D- turanose	−
16 Dulcitol	−	41 D- lyxose	−
17 Inositol	−	42 D- tagatose	−
18 Mannitol	−	43 D-fucose	−
19 Sorbitol	−	44 L-fucose	−
20 α-methyl-D- mannoside	−	45 D- arabitol	−
21 α-methyl-D- glucoside	−	46 L- arabitol	−
22 N- acetyl - glucosamine	−	47 Gluconate	−
23 Amygdalin	W	48 2-keto- Gluconate	−
24 Arbutin	W	49 5-keto- Gluconate	−

Notes: -, negative reaction; +, positive reaction; w, weak positive reaction.

**Table 2:** Biochemical and physiological characteristics of strain N1.

Biochemical reaction	Result	Biochemical reaction	Result
Gram stain	−	Alkaline phosphatase	+
Cell shape	Short rod	Esterase (C4)	+
Endospore	−	Lipoid esterase (C8)	+
Growth at 5% NaCl	+	Lipase (C14)	+
Growth at 40°C	+	Leucine aromatic aminopeptidase	+
Growth at pH 12	−	Valine aromatic aminopeptidase	+
Catalase	+	Cystine aromatic aminopeptidase	−
Oxidase	+	Trypsin	+
Arginine dihydrogenase	+	Chymotrypsin	−
Lysine decarboxylase	+	Acid phosphatase	+
Ornithine decarboxylase	−	Naphthol-AS-BI- phosphohydrolase	+
Citric acid utilization	+	α- galactosidase	+
Production of H_2_S	−	β- galactosidase	+
Urease	+	β- Glucuronidase	−
Tryptophan deaminase	−	α- glucosaccharase	+
Indole production	−	β- glucosaccharase	+
V. P. reaction	−	N- acetyl- glucosaminidase	−
Gelatinase	+	α- mannosidase	−
		β- fucosidase	−

Notes: −, negative reaction; +, positive reaction.

**Figure 1:**
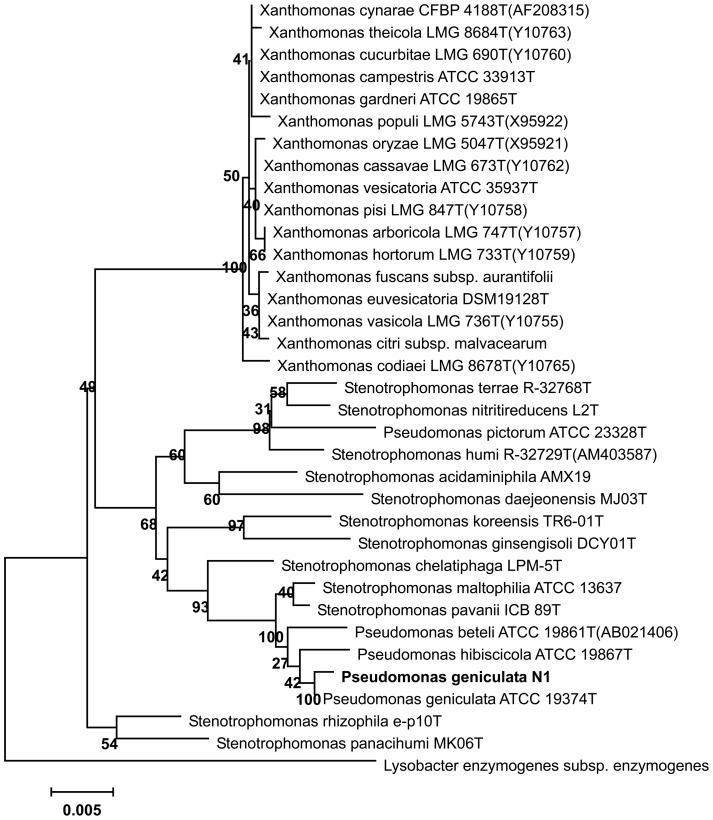
Phylogenetic tree of 16S rRNA from 35 different strains. The phylogenetic tree is constructed using the molecular evolutionary genetics analysis tool (MEGA 4.1) by neighbor joining (NJ) method [19]. The repeated bootstrapping for 1,000 times was performed.

The 16S rRNA sequence exhibits 99% identity with *Pseudomonas* and *Stenotrophomonas* species. Phylogenetic tree of 16S rRNA from 35 different strains is constructed using the molecular evolutionary genetics analysis tool (MEGA4.1) by neighbor joining (NJ) method and repeated bootstrapping for 1000 times was performed. Strain N1 is closest to the ortholog from *Pseudomonas geniculata* strain ATCC 19374T (Figure 1). In conclusion, strain N1 was classified as *Pseudomonas geniculata* based on the above results.

### Cell growth and nicotine degradation

**Figure 2:**
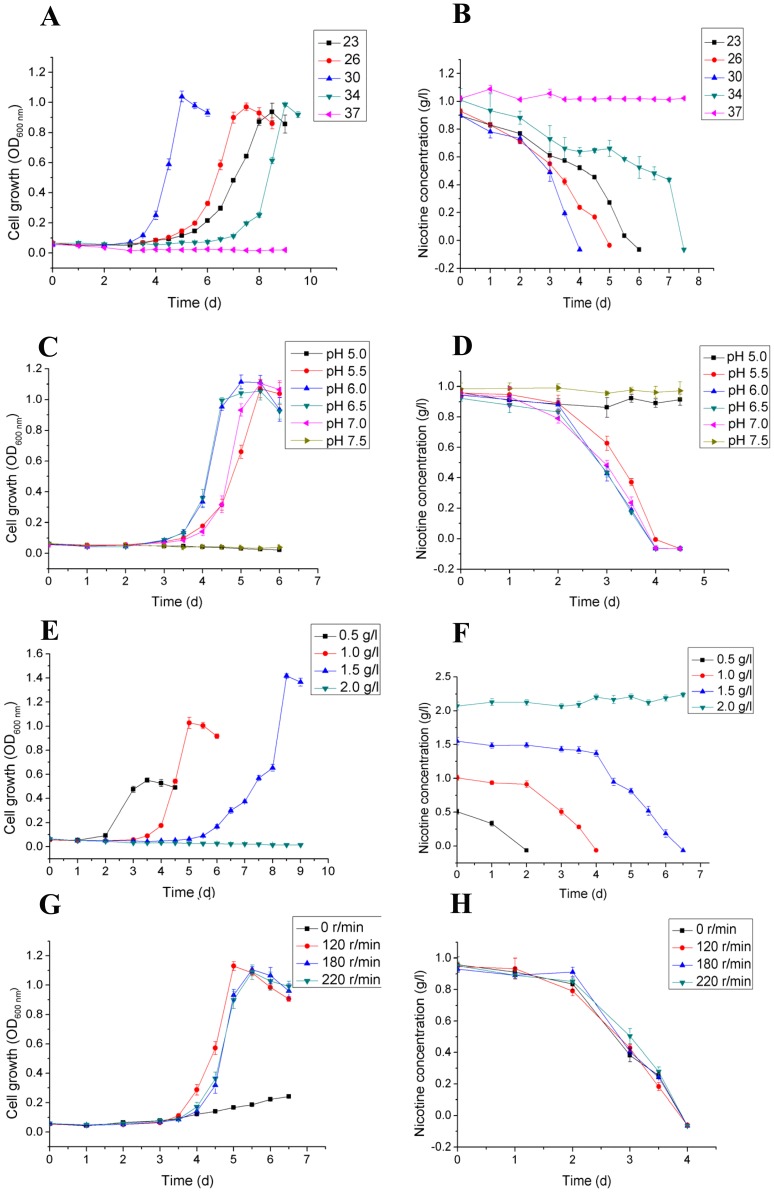
Optimization of cell growth of strain N1 in different conditions. **A**, Growth of *Pseudomonas geniculata* strain N1 at different temperatures; **B**, Nicotine degradation by strain N1 at different temperatures; **C**, Growth of strain N1 at different pH values; **D**, Nicotine degradation by strain N1 at different pH values; **E**, Growth of strain N1 at different original concentrations of nicotine; **F**, Nicotine degradation by strain N1 at different original concentrations of nicotine; **G**, Growth of strain N1 at different rotation rates; **H**, Nicotine degradation by strain N1 at different rotation rates.

The effects of temperature on strain N1 is shown in Figure 2A and 2B. Temperature has a dramatic influence on the growth of N1, which showed the maximum rate of growth and nicotine degradation at 30°C. The growth rate was much slower with a gradual drop in temperature. Notably, little-to-no growth was observed at 34°C, which indicates a narrow tolerance range for temperature.

Figure 2C and 2D show the impact of pH on the growth of strain N1. The data show that strain N1 could grow at the pH values ranging from 5.5 to 7.0. Thus, strain N1 prefers weak acidic environment, ranging from pH 6.0 to 6.5. The rate of cell growth dropped remarkably, when pH was <6.0. However, the maximum biomass did not show a major difference. With an increase in the pH value, the cell growth rate dropped slightly in a neutral environment; no growth was detected in an alkaline environment. It should also be noted that the influence of pH on nicotine degradation was not significant. The degradation rate was rather stable at pH values ranging from 6.0 to 7.5, whereas it was much slower at a pH of 5.5, which was in line with the pattern of cell growth.

Figure 2E and 2F illustrate the nicotine tolerance of strain N1. Strain N1 could grow well when the nicotine concentration was <2 g l^−1^. Moreover, with an increase in the concentration of nicotine in the growth medium, the maximum biomass increased proportionally. However, the growth was much slower when the initial nicotine concentration was 1.5 g l^−1^ rather than 1.0 g l^−1^. The maximum biomass was noted after 4.5 days, when the nicotine concentration was 1.0 g l^−1^, while it took >8 days to reach the stationary phase in the presence of 1.5 g l^−1^ nicotine.

**Figure 3:**
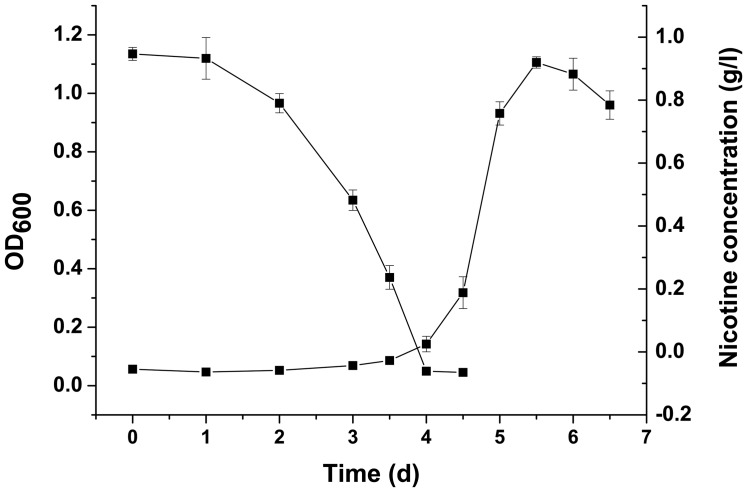
Cell growth and nicotine degradation by strain N1. Utilization of nicotine as the sole source of carbon and nitrogen by *P. geniculata* N1 for growth. Symbols: ▴, Cell growth of strain N1 at 30°C, pH 6.5 and 120 r/min; ▪‚ Nicotine concentration in the medium used for culturing strain N1. The values are the means of 3 replicates, and the error bars indicate the standard deviations.

As shown in Figure 2G and 2H, the rotary rate of the shaker can impact cell growth by altering oxygen supply. The growth was extremely slow with low maximum biomass when the growth cultures were kept stationary. This finding confirmed our initial results, which identified strain N1 as an aerobic bacterium. The optimal rotary rate of 120 rpm min^−1^ resulted in the maximum growth rate and maximum biomass production. In conclusion, *P. geniculata* N1 grows best at 30°C, pH 6.5, and 120 rpm min^−1^ with a maximum nicotine-tolerating capability of 1.5 g l^−1^ (Figure 3).

### Nicotine degradation by resting and crude cells

**Figure 4:**
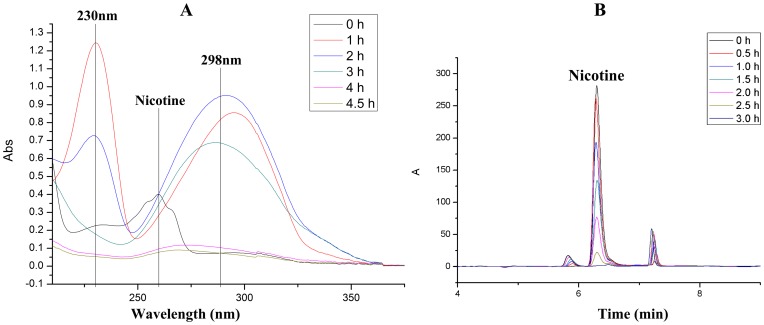
UV absorption and HPLC analysis of nicotine degradation by strain N1. **A**, UV absorption spectrum of nicotine degradation by strain N1; **B**, HPLC spectrum of nicotine metabolism by strain N1.

Resting cells harvested from nicotine medium (see materials and methods) were able to degrade 3 g l^−1^ nicotine within 3 h. As shown in Figure 4, the decrease in nicotine concentration and the formation of new peaks in the UV absorption spectrum (Figure 4A) or HPLC spectrum (Figure 4B and Figure S2) suggest the degradation of nicotine and generation of new metabolites. In contrast, the cells cultivated in LB medium did not exhibit the ability to degrade nicotine, illustrating that the enzymes required for nicotine degradation are inducible (Figure S3). The crude cells of strain N1 harvested in nicotine medium were obtained after sonication in phosphate buffer (see materials and methods). However, the cell extracts could not degrade nicotine, and it is similar to the findings with strain *Pseudomonas putida* S16 [17] (Figure S4).

### Nicotine biotransformation and metabolites identification

**Figure 5:**
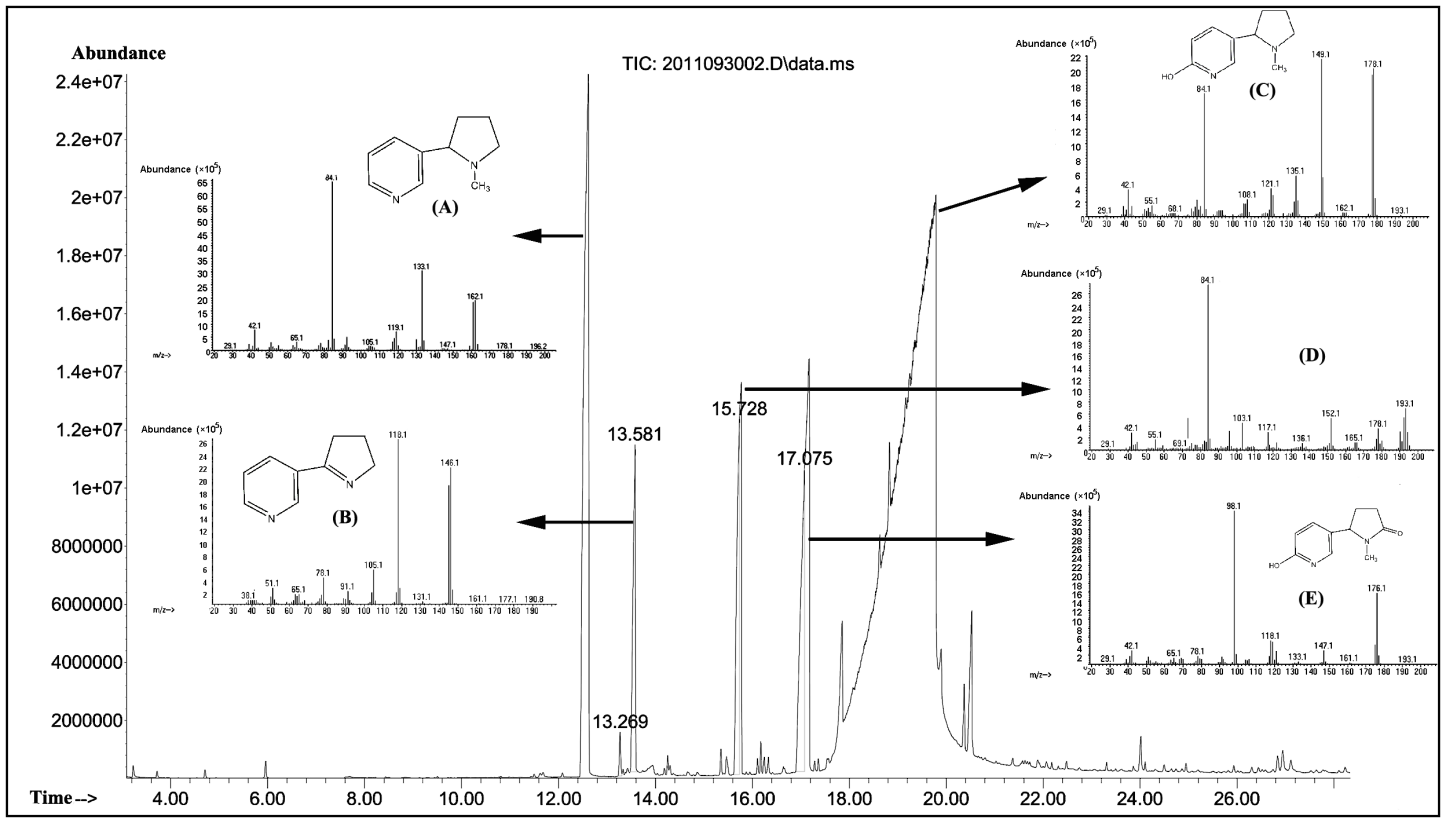
GC-MS analysis of the metabolites of nicotine degradation by strain N1. GC-MS profile and proposed structures of the intermediates of nicotine degradation by “resting cell reactions” of the strain *P. geniculata* N1. The samples were silylated by BSTFA. Compound A (nicotine, 12.603 min); compound B (myosmine, 13.581 min); compound C (6-hydroxy-nicotine, 18.883 min); compound D (trimethylsilyl (TMS) derivative of 6-hydroxy-nicotine, 15.710 min); compound E (cotinine, 17.102 min) were shown.

**Figure 6:**
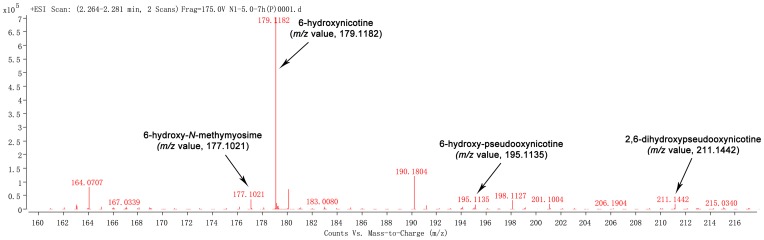
ESI-TOF-MS analysis of the metabolites of nicotine degradation by strain N1. ESI-TOF-MS analysis of the intermediates of nicotine degradation by “resting cell reactions” of strain *P. geniculata* N1. The molecular ion peaks ([M+H]^+^) of 6-hydroxynicotine (6HN, C_10_H_14_N_2_O), 6-hydroxy-*N*-methymyosime (6HMM, C_10_H_12_N_2_O), 6-hydroxy-pseudooxynicotine (6HPON, C_10_H_14_N_2_O_2_), and 2,6-dihydroxypseudooxynicotine (2,6HPON, C_10_H_14_N_2_O_3_), were shown at *m*/*z* 179.1182, 177.1021, 195.1135, and 211.1442 respectively.

The culture broth of strain N1 was yellowish green, and blue color did not develop during nicotine biotransformation. This indicated that the nicotine-degradation pathway used differed from that used by *Arthrobacter*, *Nocardioides* and *Rhodococcus* strains. The GC-MS chromatogram is shown in Figure 5. The structures of compound A (nicotine, 12.603 min), B (myosmine, 13.581 min), and E (cotinine, 17.102 min) could be identified by comparing their mass spectra with the standard GC-MS spectral library (Figure 5). Compound C (18.883 min) exhibited the following mass spectrum: 178.1 (M^+^), 177.1 ([M-H]^+^), 149.1 ([M-C_2_H_5_]^+^), 135.1 ([M-C_3_H_7_]^+^), 121.1, 108.1, 84.1 ([M-C_5_H_4_NO]^+^). The mass spectra were in partial agreement with that of previously reported 6-hydroxy-nicotine [12]. For compound D (15.710 min), its mass spectrum 193.1, 178.1, 166.1, 152.1, 117.1, 103.1, 84.1, 73, and 55.1 was determined, and was found to be identical to those of the trimethylsilyl (TMS) derivative of 6-hydroxy-nicotine. In addition, the molecular ion peaks ([M+H]^+^) of 6-hydroxynicotine (C_10_H_14_N_2_O), 6-hydroxy-N-methymyosime (C_10_H_12_N_2_O) 6-hydroxy-pseudooxynicotine (C_10_H_14_N_2_O_2_), and 2,6-dihydroxypseudooxynicotine (C_10_H_14_N_2_O_3_), were at *m*/*z* 179.1182, 177.1021, 195.1135, and 211.1442 respectively, which were identical to the calculated mass (Figure 6).

## Discussion

The highly toxic alkaloid nicotine, present in tobacco waste, is removed from the environment via mineralization by bacteria. Basic insights into the steps and intermediates of nicotine degradation by *Arthrobacter* and *Pseudomonas* species have been proposed and elucidated [4], [18], [21]. In this study, a novel nicotine-degrading bacterium N1 was isolated from tobacco leaves. The physiological and biochemical data show that the strain N1 belongs to the genus *Pseudomonas*. Most of the morphological and physiological traits of strain N1 were identical to those of *Pseudomonas geniculata* [20]. *P. geniculata* has been poorly reported in previously published literature, and this study is the first report demonstrating the nicotine-degrading ability of *P. geniculata*. In addition, it is interesting that strain N1 could utilize only a narrow range of carbon sources and efficiently degrade nicotine. Strain N1 may have a powerful membrane transport capacity; it has been reported to possess 28 multidrug efflux pump genes [22]. These fingdings imply a highly efficient nicotine uptake capacity of the strain and an efficient removal of end-products of nicotine catabolism from the cells which may help to explain the nicotine-degrading properties of strain N1.

Current understanding of nicotine degradation in bacteria is based on characterization of 6-hydroxynicotine (pyridine pathway) in *Arthrobacter* species [4] and *N*-methylmyosmine (pyrrolidine pathway) in *Pseudomonas* species [22]–[25]. Otherwise, *Agrobacterium tumefaciens* strain S33 could firstly transform nicotine to 6-hydroxy-*N*-methylmyosmine using pyridine pathway, and then further degrade 6-hydroxy-*N*-methylmyosmine to 6-hydrxoxy-3-succinoylpyridine and 2,5-dihydroxypyridine using pyrrolidine pathway [12]. In the present study, the formation of blue pigment was not observed during the transformation of nicotine by strain N1. Therefore, it can be proposed that the latter catabolic pathway of nicotine degradation is likely to be different from that of *Arthrobacter*. The intermediates 6-hydroxynicotine, 6-hydroxy-*N*-methymyosime, 6-hydroxy-pseudooxynicotine, and 2,6-dihydroxypseudooxynicotine were identified by GC-MS and LC-MS analyses. The intermediates 6-hydroxy-3-succinoylpridine and 2,5-dihydroxy-pridine in *P. putida* S16 were not detected in the “resting cell reactions” of strain N1. It can be concluded that the upper pathway of nicotine degradation in strain N1 was similar to the pyridine pathway, and the further conversion of 2,6-dihydroxypseudooxynicotine might be different from that observed in *Arthrobacter* and *Pseudomonas*. In addition, the intermediates myosime and cotinine can be detected by GC-MS. Thus, the direct demethylation to form myosime and the hydroxylation of nicotine at position 2 of pyrrolidine ring to form cotinine was proposed, and found to be similar to that of the strain *Pseudomonas* sp. CS3 [26].

In conclusion, it is proposed that the strain *Pseudomonas geniculata* N1 can decompose nicotine via a unique nicotine-degrading pathway.

## Supporting Information

Figure S1 **Characterization of** ***Pseudomonas geniculata*** **strain N1.** **A**, *Pseudomonas geniculata* N1 grown on nicotine-containing plate. **B**, Transmission electron micrograph of strain N1 cells.
(TIF)

Figure S2 **HPLC analysis of nicotine degradation by strain N1.** HPLC spectrum of metabolism of nicotine by the resting cells of strain *Pseudomonas geniculata* N1.
(TIF)

Figure S3 **Cell cultures of strain N1 in different mediums.** Nicotine concentrations in the mediums LB, LB with nicotine, and nicotine for strain *Pseudomonas geniculata* N1 growth. The values are means of three replicates, and the error bars indicate the standard deviations.
(TIF)

Figure S4 **Crude cell reactions of nicotine degradation by strain N1.** HPLC spectrum of metabolism of nicotine by crude cell reactions of strain *P. geniculata* N1.
(TIF)
