# Supplementary figures and images for: Physiological and Biochemical Characterization of a Novel Nicotine-Degrading Bacterium Pseudomonas geniculata N1
Source: PLoS One. 2014 Jan 8;9(1):e84399. doi: 10.1371/journal.pone.0084399 (PMC3885553; doi:10.1371/journal.pone.0084399)

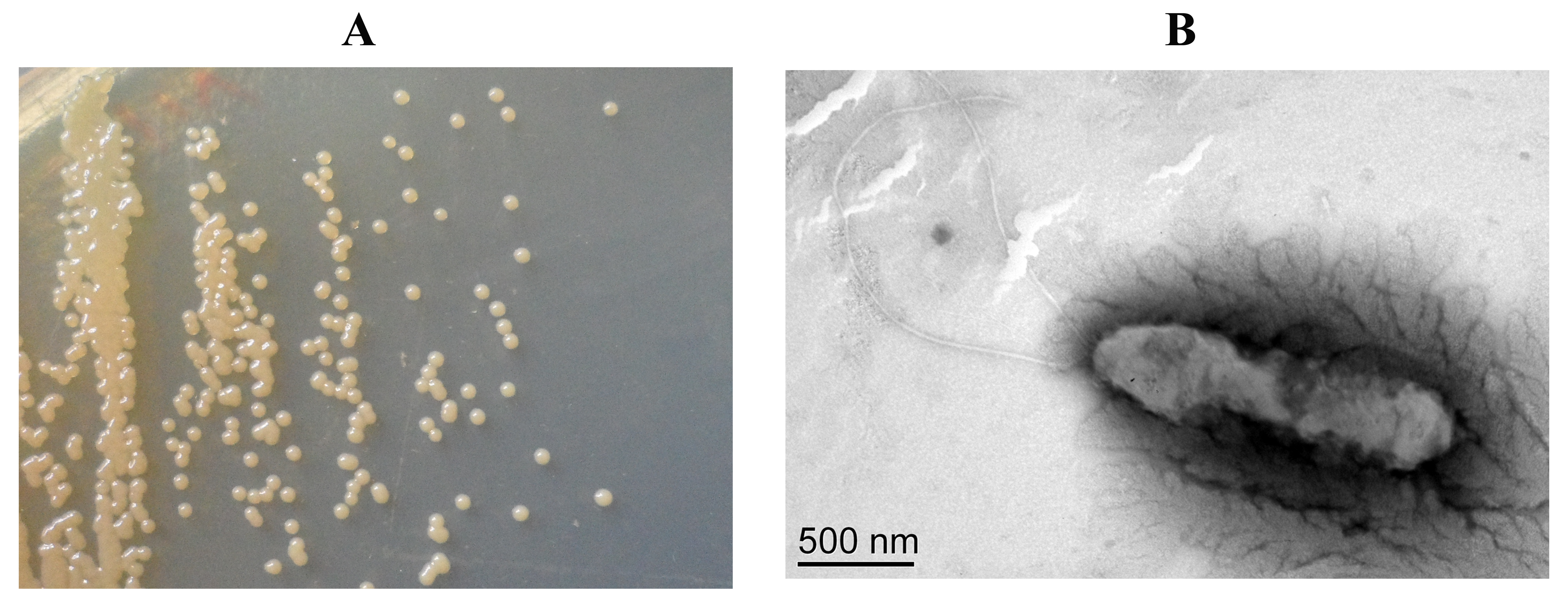

Supplement: Figure S1 — Characterization of Pseudomonas geniculata strain N1. A, Pseudomonas geniculata N1 grown on nicotine-containing plate. B, Transmission electron micrograph of strain N1 cells. (TIF) [file pone.0084399.s001.tif]

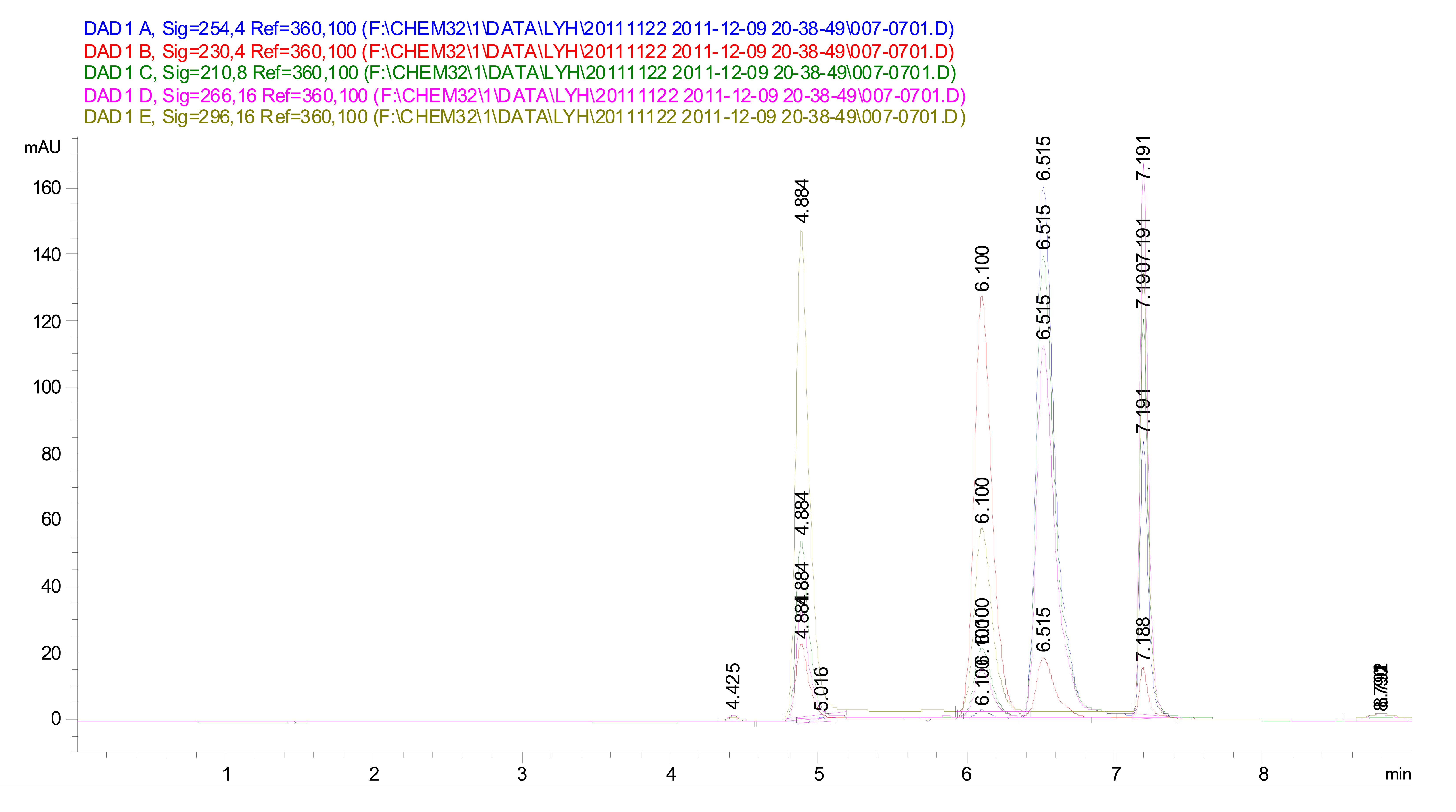

Supplement: Figure S2 — HPLC analysis of nicotine degradation by strain N1. HPLC spectrum of metabolism of nicotine by the resting cells of strain Pseudomonas geniculata N1. (TIF) [file pone.0084399.s002.tif]

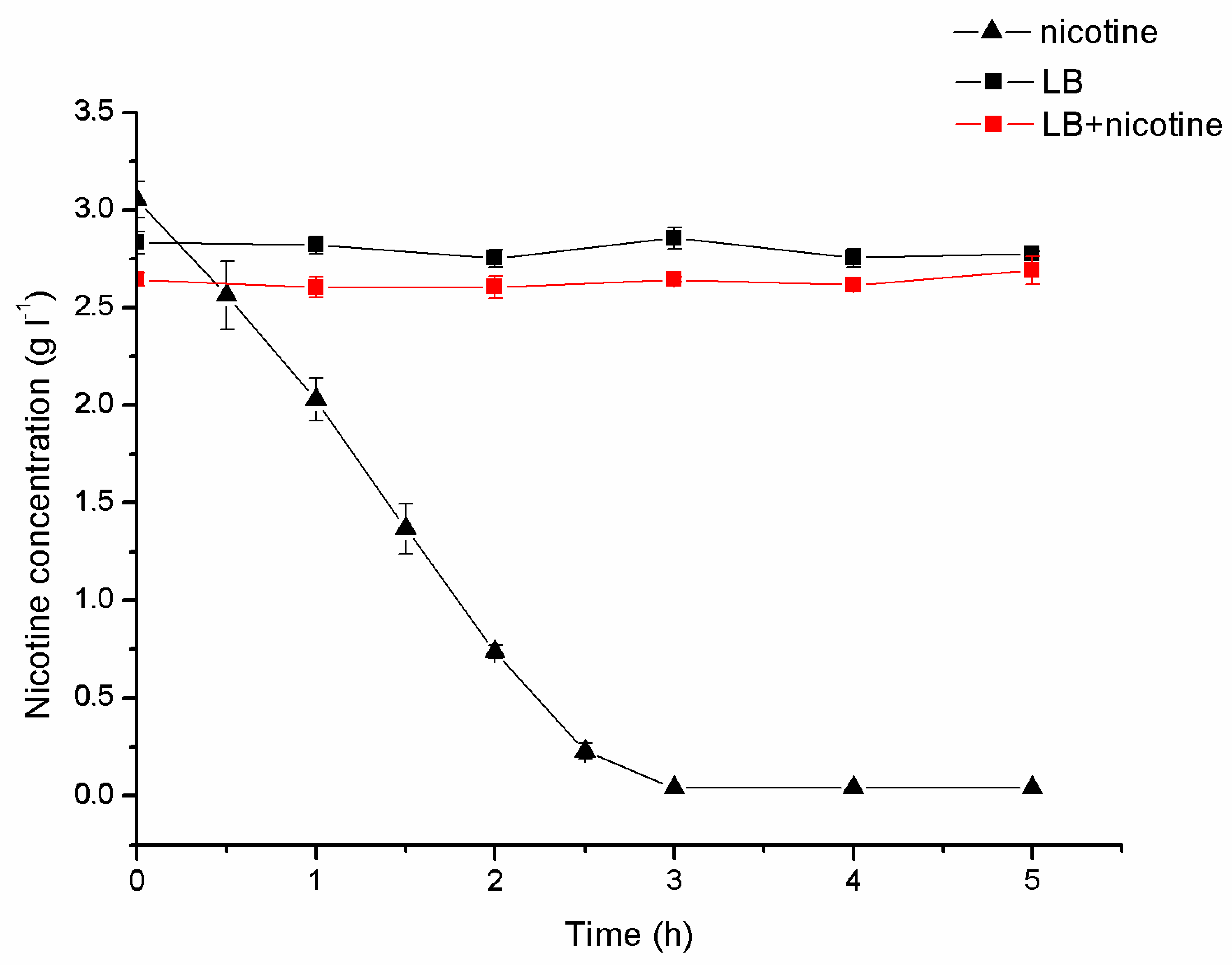

Supplement: Figure S3 — Cell cultures of strain N1 in different mediums. Nicotine concentrations in the mediums LB, LB with nicotine, and nicotine for strain Pseudomonas geniculata N1 growth. The values are means of three replicates, and the error bars indicate the standard deviations. (TIF) [file pone.0084399.s003.tif]

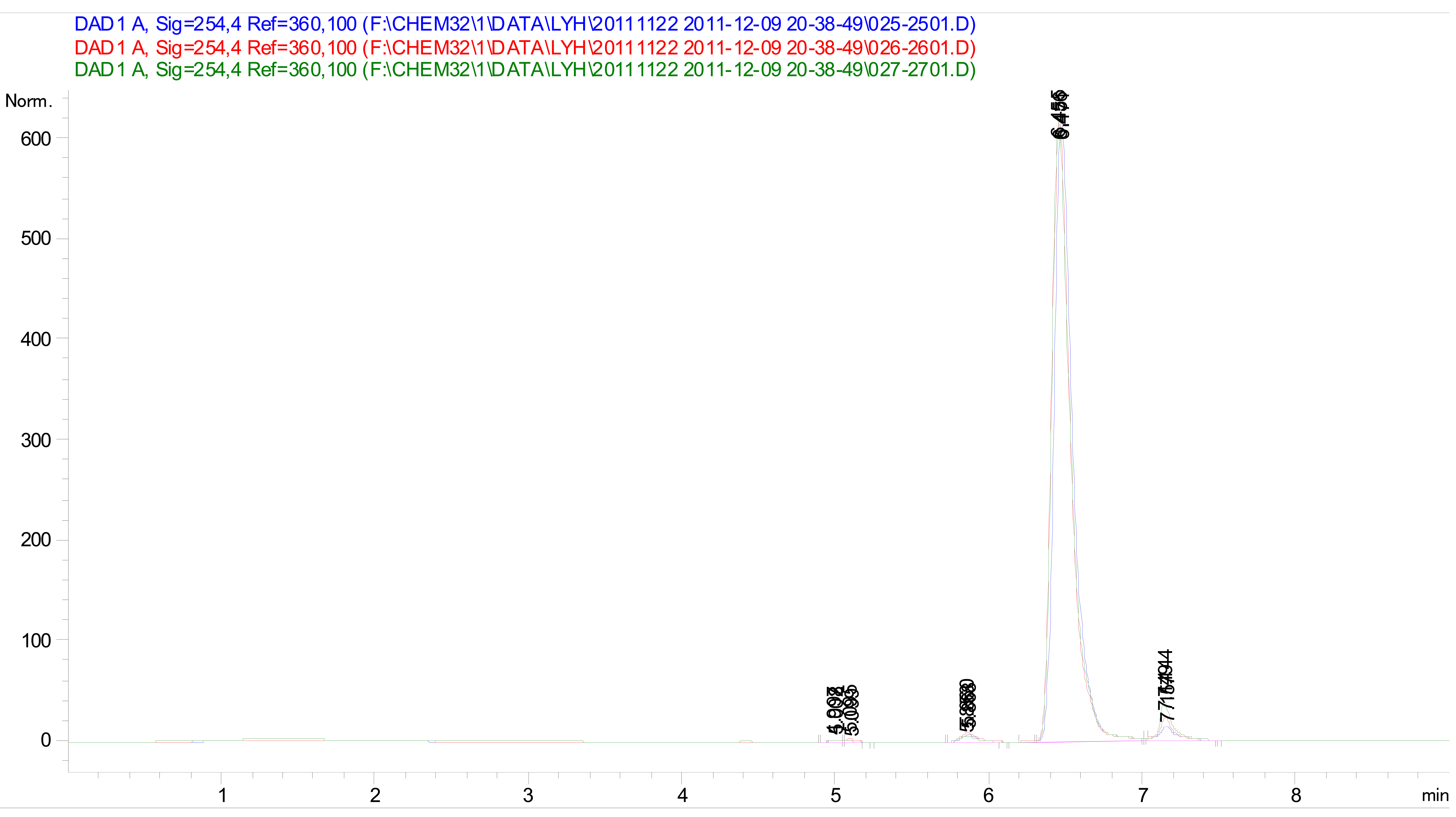

Supplement: Figure S4 — Crude cell reactions of nicotine degradation by strain N1. HPLC spectrum of metabolism of nicotine by crude cell reactions of strain P. geniculata N1. (TIF) [file pone.0084399.s004.tif]
